# Effectiveness of intra-articular autologous-conditioned serum injection in knee osteoarthritis: a meta-analysis study

**DOI:** 10.2144/fsoa-2021-0069

**Published:** 2021-10-11

**Authors:** Seyed A Raeissadat, Seyed M Rayegani, Mohammad R Sohrabi, Nafisseh Jafarian, Mohammad N Bahrami

**Affiliations:** 1 Physiatrist, Associate Professor, Physical Medicine & Rehabilitation Department & Research Center, School of Medicine, Shahid Beheshti University of Medical Sciences, Tehran, Iran; 2 Physiatrist, Professor, Physical Medicine & Rehabilitation Department & Research Center, School of Medicine, Shahid Beheshti University of Medical Sciences, Tehran, Iran; 3 Community Medicine Specialist, Professor, Department of Community Medicine, School of Medicine, Shahid Beheshti University of Medical Sciences, Tehran, Iran; 4 Physiatry Resident, Physical Medicine & Rehabilitation Department & Research Center, School of Medicine, Shahid Beheshti University of Medical Sciences, Tehran, Iran; 5 Orthopedic Surgeon, Pediatric Orthopedics Fellowship, Orthopedic Surgery Department & Research Center, School of Medicine, Shahid Beheshti University of Medical Sciences, Tehran, Iran

**Keywords:** autologous conditioned serum, intra-articular injection, knee osteoarthritis, meta-analysis, systematic review

## Abstract

**Aim:**

Knee osteoarthritis is a common disabling disorder, with no curative treatment. This study aims to assess autologous conditioned serum effectiveness in its treatment.

**Materials & methods:**

Following a systematic search (2000–2020) on major databases and screening and filtering processes, eight articles were included in the final analyses. The pooled effect of visual analog scale (VAS) and the Western Ontario and McMaster Universities (WOMAC) variables was evaluated before and after the intervention.

**Results:**

The combined standardized mean difference for the global WOMAC score was -2.44 and the combined weighted mean difference was -22.92. The combined standardized mean difference for the VAS score was -3.77 and the combined weighted mean difference was -32.37 (p < 0.000).

**Conclusion:**

This meta-analysis reported that the autologous conditioned serum can reduce pain and improve function (VAS and WOMAC outcome measures) in patients with knee osteoarthritis.

## Lay abstract

Knee osteoarthritis is a common and disabling disease with various therapeutic options; regenerative treatment among them is a large group of autologous blood products with different contents of growth signaling or anti-inflammatory components. Autologous conditioned serum (ACS) is one of the recently introduced choices gaining attention. The studies on ACS reported mostly promising but mixed results, this study aimed to pool the available data on the subject to obtain a conclusion on the effectiveness of ACS in knee osteoarthritis, and it resulted in the favor of this method, in reducing pain and improving function.

Osteoarthritis (OA) as the most common arthritis affecting around 250 million people worldwide, is a chronic, disabling condition in which joint inflammation, cartilage breakdown and bone remodeling contribute to a syndrome of chronic pain, stiffness and impaired movement [1–3]. Knee and hip joints are the most susceptible weight-bearing joints in developing OA [3]. The elderly (~35% of patients over 65 years old) females, patients with obesity and African–Americans are at the highest risk [2]. The prevalence of OA varies widely between countries, from 2.8% in the Philippines to 19% in rural regions of Iran [4–6].

The incurable nature of this chronic, progressive and disabling disease and also limited treatment choices to reduce its symptoms impose a significant socioeconomic burden on the healthcare systems internationally [7]. The current treatment options are mostly applied to control the symptoms and improve the quality of life. These include nonpharmacological treatments such as exercise, aqua-therapy and physiotherapy and pharmacological treatments (Non-Steroidal Anti-Inflammatory Drugs, opioids, serotonin norepinephrine reuptake inhibitors, etc.) and therapeutic interventions including intra-articular injections of steroids, hyaluronic acid, regenerative medicine treatments such as mesenchymal cell products and platelet-rich plasma (PRP). As a widely accepted and applied treatment, PRP is a platelet-rich plasma product, mainly classified in four families, based on their fibrin architecture and cell content: pure PRP; leukocyte- and PRP; pure platelet-rich fibrin; and leukocyte- and platelet-rich fibrin; each presenting different biological signatures and mechanisms, and clinical applications [8]. Despite their common acceptance, these options though have not been proven to be effective in halting the disease progression [2,9–12].

On the other hand, surgical interventions such as arthroscopic lavage and debridement, cartilage repair and total knee replacement are also available; although many factors (patient’s age, co-morbidities, socioeconomic status, etc.) are to be considered in choosing these methods and their effectiveness [13]. In addition to the degenerative process, inflammation is also known to contribute to the pathophysiology of the disease. The inflammatory cytokines IL-1β and TNF-α play a critical role in driving the progression of OA, causing pain and also cartilage degeneration (IL-1 particularly inhibits synthesis of the matrix and also promotes its degradation). Therefore, treatment options that inhibit inflammatory signaling pathways may be effective in slowing the disease progression [14,15].

Intra-articular injection of autologous conditioned serum (ACS) is one of the newly emerged regenerative therapeutic options, which is shown to be improving clinical symptoms in equine studies [15]. This product (introduced in the 1990s as Orthokine [Orthogen, Düsseldorf, Germany]) is obtained by incubating whole blood with CrSO4-coated glass beads at 37°C; the process stimulates the synthesis of the IL-1 receptor antagonist (IL-1ra) and other anti-inflammatory cytokines, such as IL-4, IL-10 and IL-13. Therefore, this method might have a beneficial effect on the symptoms and disease progression of OA [16,17]. After incubation, the next steps are centrifugation and filtration procedures [18]. It is kept at -20°C and injected into the joint at 1-week intervals [18,19].

Although during the last two decades various studies have suggested ACS as an effective and safe treatment option for knee OA [1,3,13,16,17,20]; some researchers have not thoroughly confirmed this conclusion [21,22]. Regarding the mentioned heterogeneity of the research on this topic (different methods, various patients and diverse results), conducting a systematic review and meta-analysis to aggregate and integrate the results of the presently available data is of practical application to make a conclusive clinical decision.

## Methods

### Protocol & registration

This systematic review and meta-analysis aimed to investigate the effectiveness of intra-articular ACS injection in patients with knee OA. The protocol was submitted and registered in the PROSPERO database (ID: CRD42019146738) available at: https://www.crd.york.ac.uk/PROSPERO/display_record.php?RecordID=146738.

PRISMA 2009 checklist was used to guide the methods of this review [23].

### Eligibility criteria

Two reviewers (an orthopedic specialist and a physiatry resident) independently evaluated the titles and abstracts of all identified studies to find potentially relevant articles. The studies were eligible for the analysis if they met all of the following inclusion criteria:

- Interventional studies (clinical trials)
- Investigated knee OA in adult patients (older than 18 years), diagnosed by a clinical physician according to the American College of Rheumatology or Kellgren–Lawrence radiologic index
- Examined the effectiveness of intra-articular ACS injection for the treatment of knee OA
- Human clinical trials
- Reported extractable outcome data for at least one measure of pain or function or stiffness by visual analog scale (VAS) or the Western Ontario and McMaster Universities (WOMAC) questionnaire

The exclusion criteria were:

- Duplicate publications
- Improper study design (reviews, case reports, etc.)
- Studies with unavailable full text
- Studies with insufficient presented data
- Subjects with any history of prior knee surgery or trauma, inflammatory or systematic diseases
- Animal model clinical trials
- *In vitro* studies

### Information sources

An extended systematic search was performed by the two independent researchers (MN Bahrami and N Jafarian) on electronic and nonelectronic sources. Major electronic databases including PubMed (NLM), Cochrane Library (CENTRAL), Science Direct, Scopus, Web of Science, Embase and LILAC, as well as search engines like Google Scholar. An expert librarian assisted in performing a manual search in nonelectronic bibliographic sources.

The gray literature was investigated through websites such as http://opengrey.eu/search/ and https://www.ntis.gov/; contacting frequent authors on the subject and the relevant companies and incorporations. Also, websites of https://www.irct.ir, https://www.who.int/ictrp/search/en/ and https://clinicaltrials.gov/ were searched for possible ongoing clinical trials.

### Search

The information sources were searched for published and unpublished documents in the timeframe of January 2000 up to September 2020, with no limits applied for language or country to maximize the sensitivity.

The search queries included combined word text with truncations, wildcard operators (‘*’), proximity and Boolean operators (‘AND’, ‘OR', ‘NOT', ‘NEAR', ‘ADJ’), as well as the specific controlled vocabulary for each database if available (e.g., MeSH terms in PubMed).

The search strategy contained different combinations of the following keywords: ‘autologous conditioned serum', ‘autologous conditioned plasma', ‘ACS', ‘Interleukin-1 receptor antagonist', ‘IRAP', ‘Orthokine', ‘knee', ‘osteoarthritis', ‘intra-articular injection', ‘treatment'.

### Study selection

Two reviewers (MN Bahrami and N Jafarian) independently searched the electronic and nonelectronic databases and decided which study to be included. All results were collected using a reference manager software, EndNote ver. X8, to manage the search results and remove duplications. The primary search results were screened by title and abstract after deduplication. Relevant papers were chosen according to the above-mentioned inclusion and exclusion criteria. Any disagreement between the two reviewers (N Jafarian and MN Bahrami) was discussed with a senior author (SA Raeissadat or SM Rayegani).

### Data collection process & data items

Data were extracted from eight articles into a previously piloted data extraction spreadsheet, designed in Microsoft Excel version 2013 by the two researchers. Any disagreements were solved with discussion or referring to the senior author. The form included the following information: study ID, author’s first name, year of publication, country, study design, patients’ demographic data such as mean age, mean BMI, presenting symptoms and severity grade, study sample size, randomization method, number of patients in each treatment or the control group, doses, and intervals of injections, blind or guided injections, follow-up intervals, outcome measures and adverse reactions.

According to the above-mentioned inclusion criteria, the outcome measures were extracted in the datasheet including pain or stiffness or function evaluated by VAS or WOMAC scoring systems before and after the intervention in the following assessments.

### Risk of bias in individual studies

The risk of bias of the included studies was assessed by two reviewers (N Jafarian and MN Bahrami, while supervised by SA Raeissadat and SM Rayegani), following the methods recommended by the Cochrane collaboration [24] using RevMan software 5.3. The included trials were evaluated in the following categories: selection bias (random sequence generation and allocation concealment), performance bias (blinding of participants and personnel), detection bias (blinding of researchers conducting outcome assessments), attrition bias (incomplete outcome data), reporting bias (selective reporting) and other sources of bias. A judgment of ‘low risk', ‘high risk’ or ‘unclear risk’ of bias was provided for each domain. Any disagreement was solved by discussion or by senior authors.

### Summary measures & synthesis of results

Statistical analyses were performed by using the STATA 15.0 (StataCorp LLC, TX, USA). Standardized mean difference (SMD) and weighted mean difference (WMD) were used to evaluate the pooled effect of VAS and WOMAC variables before and after the intervention. To calculate heterogeneity among studies, we used the Chi-squared test and the I^2^ index. The results were weighted by the Glass (g) method, the random effect model was used and the overall effect size was displayed in the form of a forest plot diagram.

### Risk of bias across studies

The possibility of publication bias was evaluated by a funnel plot for asymmetry, which can result from the nonpublication of small trials with negative results; considering that some other factors, such as different qualities of the trials or true heterogeneity, can cause asymmetry in the funnel plot.

### Additional analyses

According to the data extracted and the outcome measures, subgroup analysis of the WOMAC scores was performed based on the follow-up periods on studies with similar variables to obtain a better comparison.

## Results

### Study selection & characteristics

**Figure 1.**
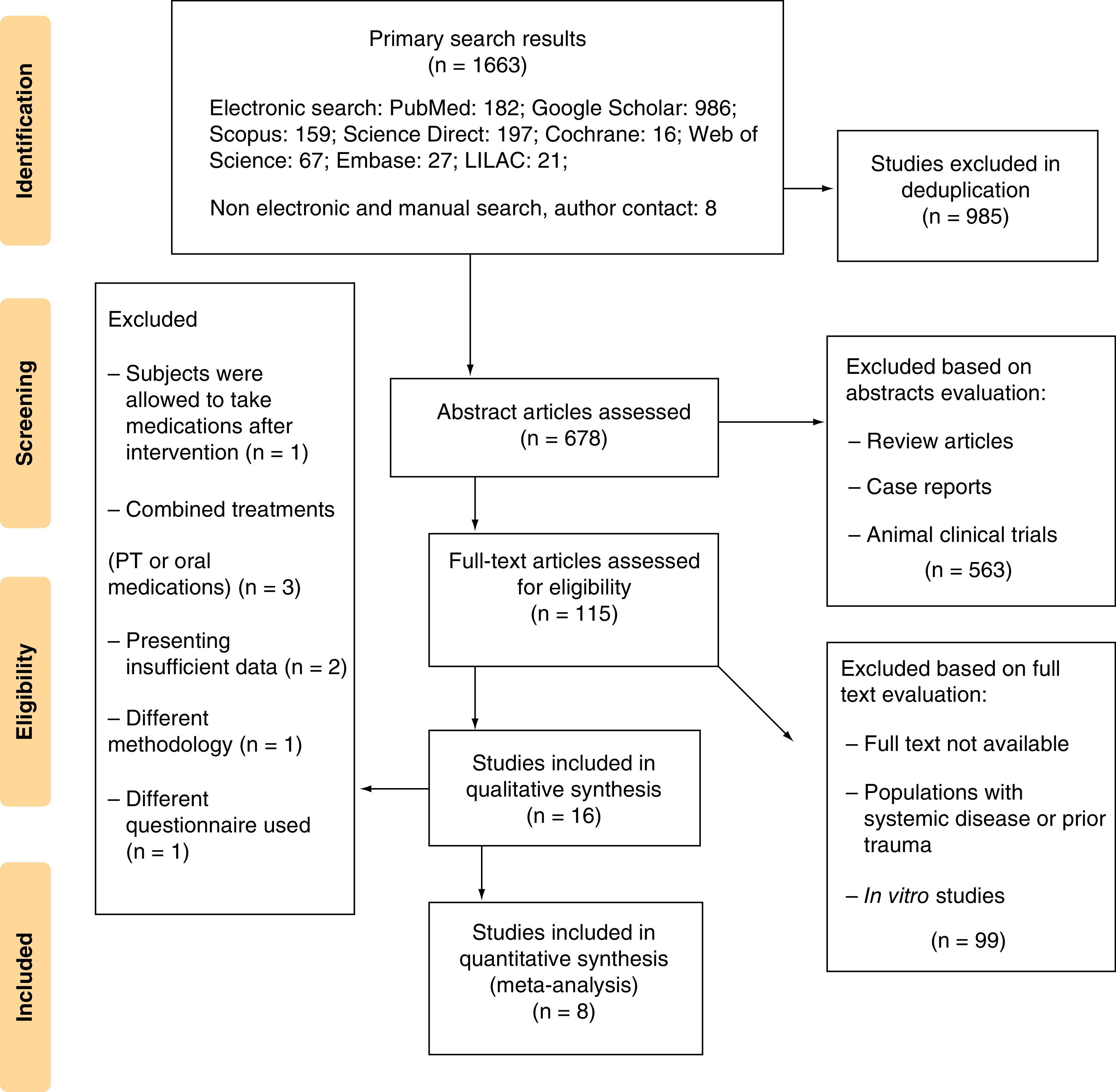
PRISMA flow diagram showing selection process of included studies.

A total number of 1663 papers were recruited in the primary search by two separate search processes; after deduplication, 678 studies were screen based on their title and abstract and those with improper study design (reviews, case reports, etc.) or with animal model subjects were excluded. The remaining 115 pieces of research were assessed thoroughly, for exclusion criteria in population or intervention or outcome measures of the studies (e.g., excluding those with combinational therapeutic interventions or subjects with a history of prior knee trauma or surgery, as well as *in vitro* trials). The studies with insufficient details or unavailable full text (despite contacting the authors) were excluded as well. Of the 16 remaining articles, eight papers were finally considered eligible to enter the meta-analysis (Figure 1).

Three studies were excluded because of a combination of treatment with physiotherapy [25] and analgesic medication use [20,26]. One study had a different methodology from those we were considering and did not use a questionnaire to assess patients [27]. Furthermore, in this study, individuals who continued to take their analgesic drugs during and after treatment were not isolated [27]. Two studies were omitted due to insufficient statistical information on the variables under examination [21,28]. One study was also excluded due to the use of different questionnaires [18].

The whole process of study screening and selection was carried out by two researchers, N Jafarian and MN Bahrami, and closely supervised by senior authors experts in the field, SA Raeissadat and SM Rayegani.

### Risk of bias within studies

**Figure 2.**
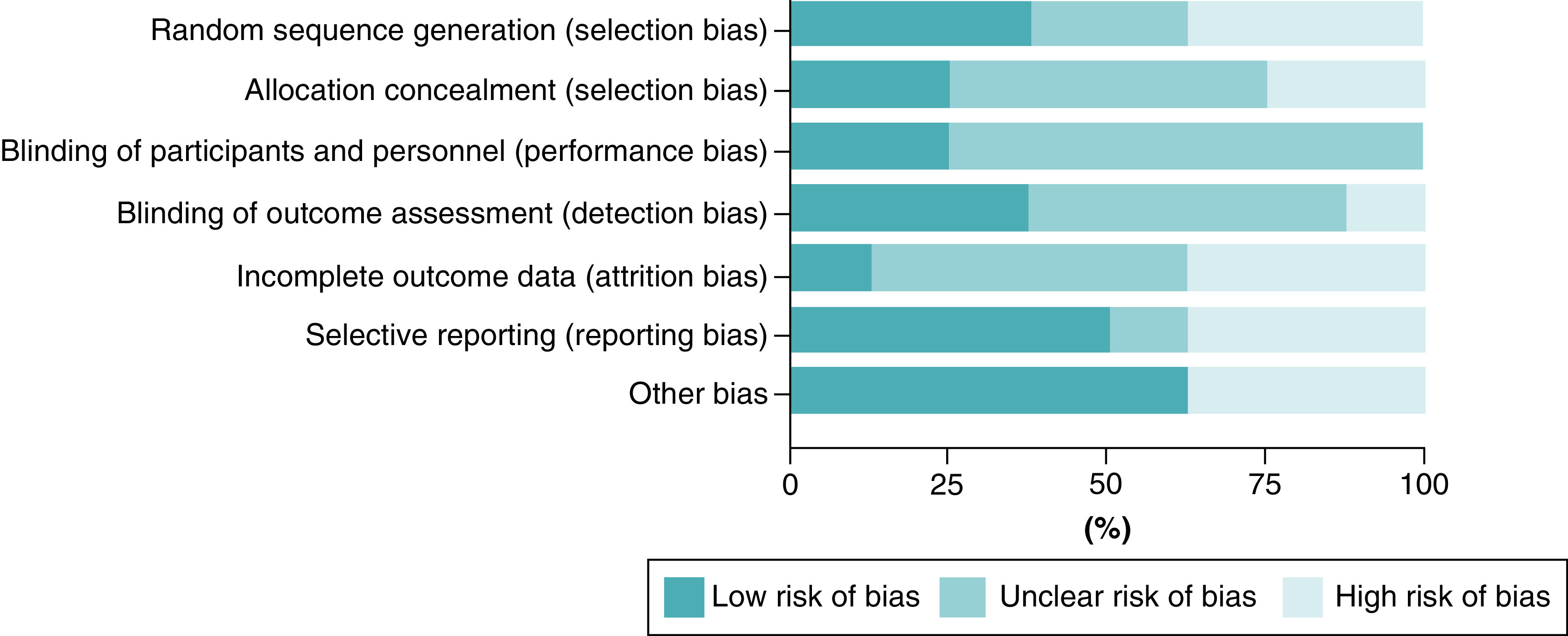
Risk of Bias.

**Figure 3.**
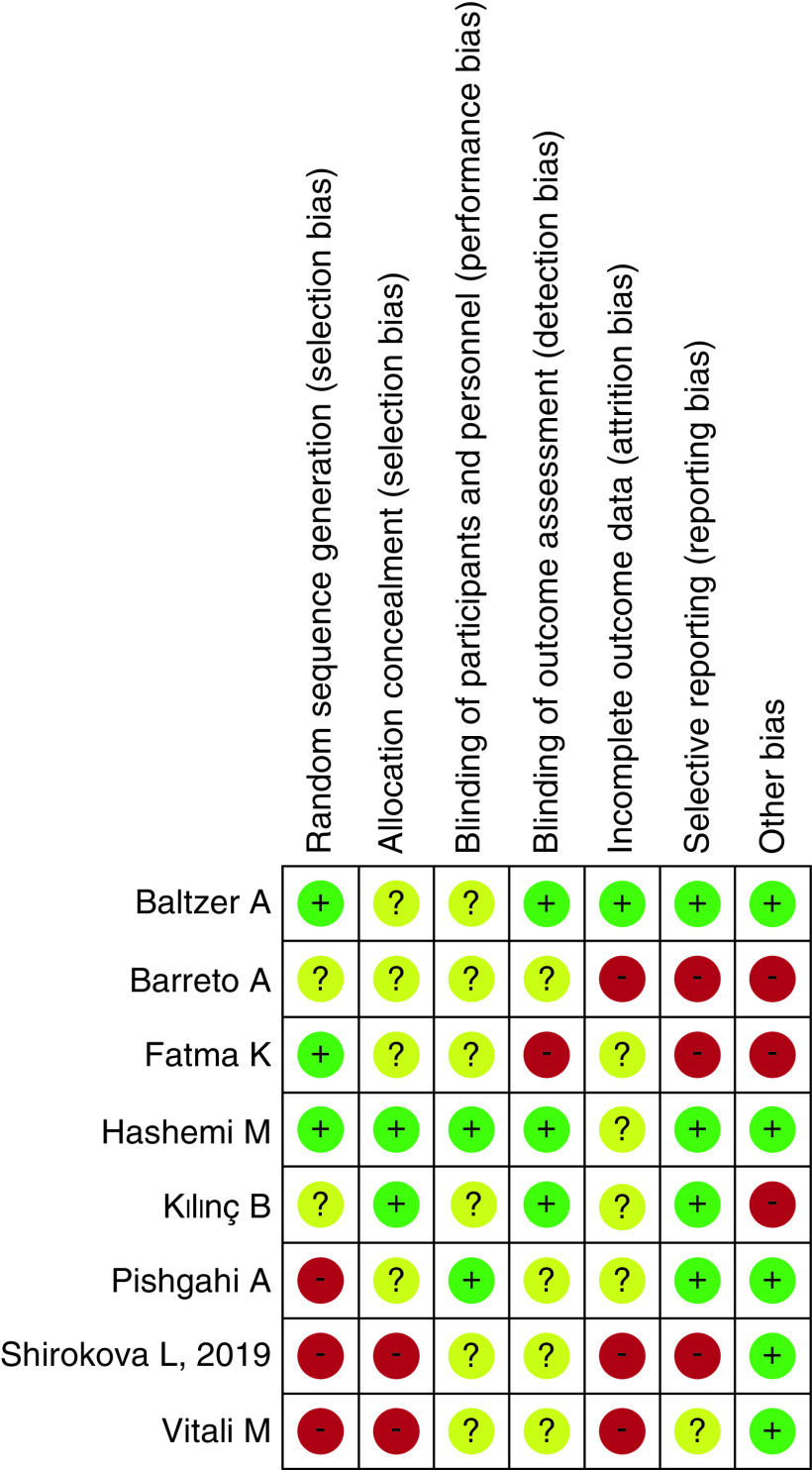
Risk of bias summary; estimated risk of bias item for each included study.

As explained in the Methods section, the risk of bias was evaluated according to the Cochrane risk of bias tool by two researchers and confirmed by the senior author, six studies were considered as having an acceptably low risk, and two with a high risk of bias (detailed results in Figures 2 & 3).

### Results of individual studies

#### Summary of studies

The present study included the final eight papers published between 2009 and 2020, with a total number of 439 patients and at least 3 months of follow up. These studies were conducted in various countries: the USA, Germany, Italy, Iran, Russia, Egypt and Turkey. The combined mean age of the subjects was 58.68 years (95% CI: 57.18–60.17) and the mean BMI was 32.20 (95% CI: 30.50–33.90). The main characteristics of these studies are summarized in a table presented as Table 1.

**Table 1.** Characteristics of the included studies.

Study	Year	Country	Type of study	Sample size	Age (mean ± SD)	Sex ratio (female/male)	BMI (kg/m^2^; mean ± SD)	Follow-up duration (months)	ACS dose	Injections (n)
Baltzer *et al.*	2009	Germany	RCT	134	53.8 ± 12.2	65/69	-	6	2 cc/twice a week	6
Fatma *et al.*	2014	Egypt	Prospective study	30	54.21 ± 5.95	17/13	-	3	1 cc/weekly	3
Barreto and Braun	2017	USA	RCT	100	61.2 ± 1.2	68/32	33.8 ± 1.4	12	1 cc	6
Kılınç and Öç	2019	Turkey	RCT	33	57.66 ± 8.21	19/14	-	12	?cc/twice a week	6
Shirokova *et al.*	2019	Russia	nRCT	26	56.6 ± 11	26/0	32.4 ± 4.6	3	2.5 cc/twice a week	6
Shirokova *et al.*	2019	Russia	nRCT	39	61.2 ± 8.4	39/0	31.2 ± 5.2	3	2.5 cc/twice a week	6
Hashemi *et al.*	2020	Iran	RCT	30	56.8 ± 8.6	16/14	31.1 ± 3.4	6	2 cc/weekly	4
Pishgahi *et al.*	2020	Iran	RCT	32	61.28 ± 1.67	20/12	-	6	2 cc/twice a week	6
Vitali *et al.*	2020	Italy	RCT	15	63.5	-	-	6	? ml / weekly	4

?: Dosage is not reported; ACS: Autologous conditioned serum; RCT: Randomized clinical trial; SD: Standard deviation.

##### Baltzer *et al.*

This study was a double-blinded randomized controlled trial, with 376 knee OA patients assigned randomly in three groups of interventions to compare the effectiveness of ACS, hyaluronic acid (HA) and saline as a placebo. The ACS group contained 134 patients with a mean age of 53.8 ± 12.2 years, after receiving six doses of 2 ml ACS twice a week, they were monitored for 6 months with VAS and WOMAC scores. The data demonstrated a significant improvement in outcome measures of pain and function in the ACS group, although the disease-modifying feature of this product was not concluded.

##### Fatma *et al.* [30]

This prospective study on a total number of 30 knees with OA (mean age: 54.21 ± 5.95 years) was designed with 3 weekly intra-articular injections of 1 ml ACS and 3 months follow up with the WOMAC score. Their results revealed a significant improvement in the outcome measure, which persisted for 3 months and suggested ACS as a reliable treatment for knee OA.

##### Barreto and Braun

In this randomized clinical trial (RCT), 100 patients (mean age 61.2 ± 1.2 years) with knee OA were treated with six doses of 1 ml ACS (intra-articular ultrasound-guided injections) and the outcome measures of VAS and Extra Short Musculoskeletal Functional Assessment (XSMFA-D) and Patient Global Impression of Change survey were evaluated after 12 months of follow up. Despite the gender inequality in the subjects of this study (female/male: 68/32) and the BMI of 33.8 ± 1.4, the data showed pain reduction and enhanced quality of life experienced by patients. ACS was introduced as a safe and effective option for patients with knee OA.

##### Kilinc and Öç

This RCT recruited 33 patients with knee OA (mean age of 57.66 ± 8.21 years) and monitored them for 12 months after six doses of ACS injections twice a week. The outcome measures of VAS, Knee Injury and Osteoarthritis Outcome Score (KOOS) and Knee Society Score were assessed and resulted in significant improvement. Hence, the authors suggested ACS as an effective treatment.

##### Shirokova *et al.*

This clinical, nonrandomized trial compared ACS versus PRP in knee OA with subclinical or moderate synovitis symptomology in 123 patients with knee OA, Kellgren–Lawrence grade II–III. The ACS group included 65 female patients who were monitored for 3 months after six intra-articular injections of 2.5 ml ACS twice a week. Since the subjects of this study were further subdivided into two groups of subclinical synovitis and moderate synovitis, this study was introduced as two separate data collections in the meta-analysis to minimize possible bias. The evaluation of outcome measures (VAS and WOMAC) showed significant improvement and the authors concluded that ACS is superior to PRP in improving joint homeostasis in this female knee OA patient cohort. Although the lack of blinding and gender inequality in this study requires cautious interpretation of the results.

##### Hashemi *et al.*

In this RCT, 60 patients of knee OA (mean age: 56.8 ± 8.6 years) were randomly allocated in two groups of 30, treated with either four doses of 2 ml ACS weekly injections, or three doses of 2 ml HA as the control group. Pain, symptoms, daily activities, sport recreational performance and knee-related quality of life were assessed via KOOS and WOMAC scores in 6 months of follow up. This study reports significant improvement in the ACS group and suggested this method as a safe and effective option for knee OA patients.

##### Pishgahi *et al.*

This RCT included 92 patients with knee OA to compare the therapeutic effects of ACS (n = 32), dextrose prolotherapy (n = 30) and PRP (n = 30). The prolotherapy group received three weekly doses of a combination of 50% dextrose (2 ml), bacteriostatic water (2 ml) and 2% lidocaine (1 ml) intra-articular ultrasound-guided injections. The PRP and ACS group were treated with twice-weekly injections. The results of VAS and WOMAC scores after 6 months of follow up showed significant improvement in ACS and PRP groups, with the superiority of ACS. The authors mentioned ACS as an effective and tolerable treatment choice for knee OA and regarding less variability in its processing and less reported side effects (compared with PRP), it can be suggested as an alternative option.

##### Vitali *et al.*

In this RCT with 15 patients of knee OA (mean age: 63.5 years), after four landmark-based intra-articular injections of ACS, patients were followed up for 6 months and Knee Society Score, VAS, and WOMAC scores were evaluated. Despite notable mean age differences in male/female subgroups (57.1/69 years old, respectively), this study resulted in considerable improvement in pain and joint function outcome measures with no significant adverse effects.

### The overall adverse events profile

Out of these eight studies, three of them (Kilinc and Öç, Pishgahi *et al.* and Shirokova *et al.*) reported no adverse events in the subjects; on the other hand, Vitali *et al.* mentioned a few numbers of side effects: five patients with local pain, three with joint swelling and one with rigidity. The study by Baltzer *et al.* also reported adverse events in the three groups of their study: two out of 107 subjects in the saline group, five out of 135 in the hyaluronic acid group and none in the ACS group showed symptoms of local side effects; four cases with joint effusion which was aspirated and evaluated (no crystals or infection detected) and one case of skin reaction in the HA group. All of these symptoms were improved in a few days. In the ACS group, only mild-to-moderate local symptoms were reported that resolved in few hours requiring no further intervention.

The frequency of adverse events was not reported in the other three papers (Hashemi *et al.*, Barreto and Braun, and Fatma *et al.*).

This overview suggests a low-risk profile for adverse events in the ACS therapy.

### Synthesis of results

#### Summary of pooled results

##### Global WOMAC score

**Figure 4.**
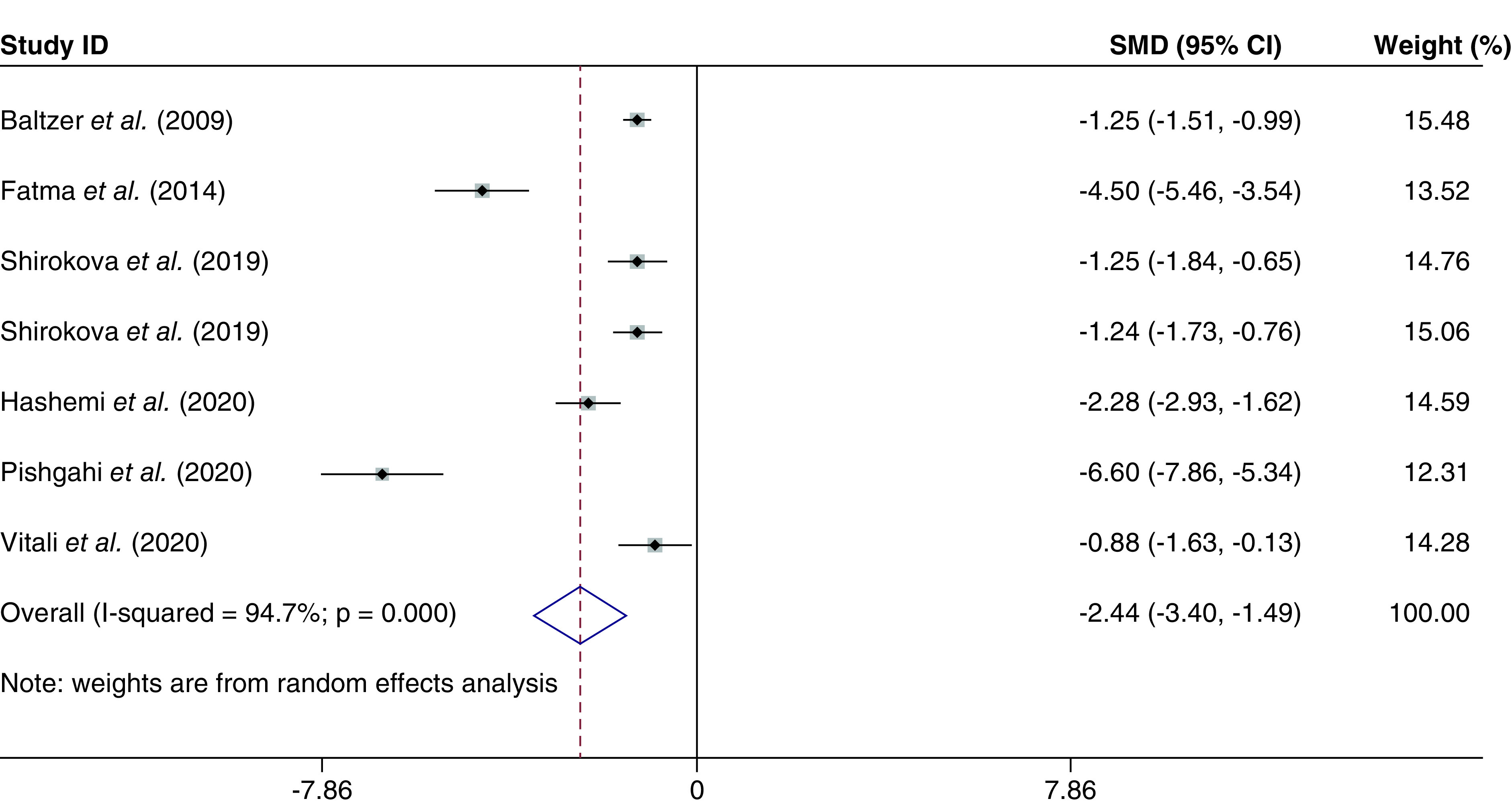
The global Western Ontario and McMaster Universities score based on the random-effected model.

The global WOMAC score was evaluated in six studies [29–34], with a total of 306 individuals. The combined SMD for the global WOMAC score was -2.44 (95% CI: -3.40 to -1.49; p < 0.000; I^2^ = 94.7%) (Figure 4). In addition, combined WMD was -22.92 (95% CI: -28.22 to -17.63; p < 0.000; I^2^ = 89.0%).

##### VAS score

**Figure 5.**
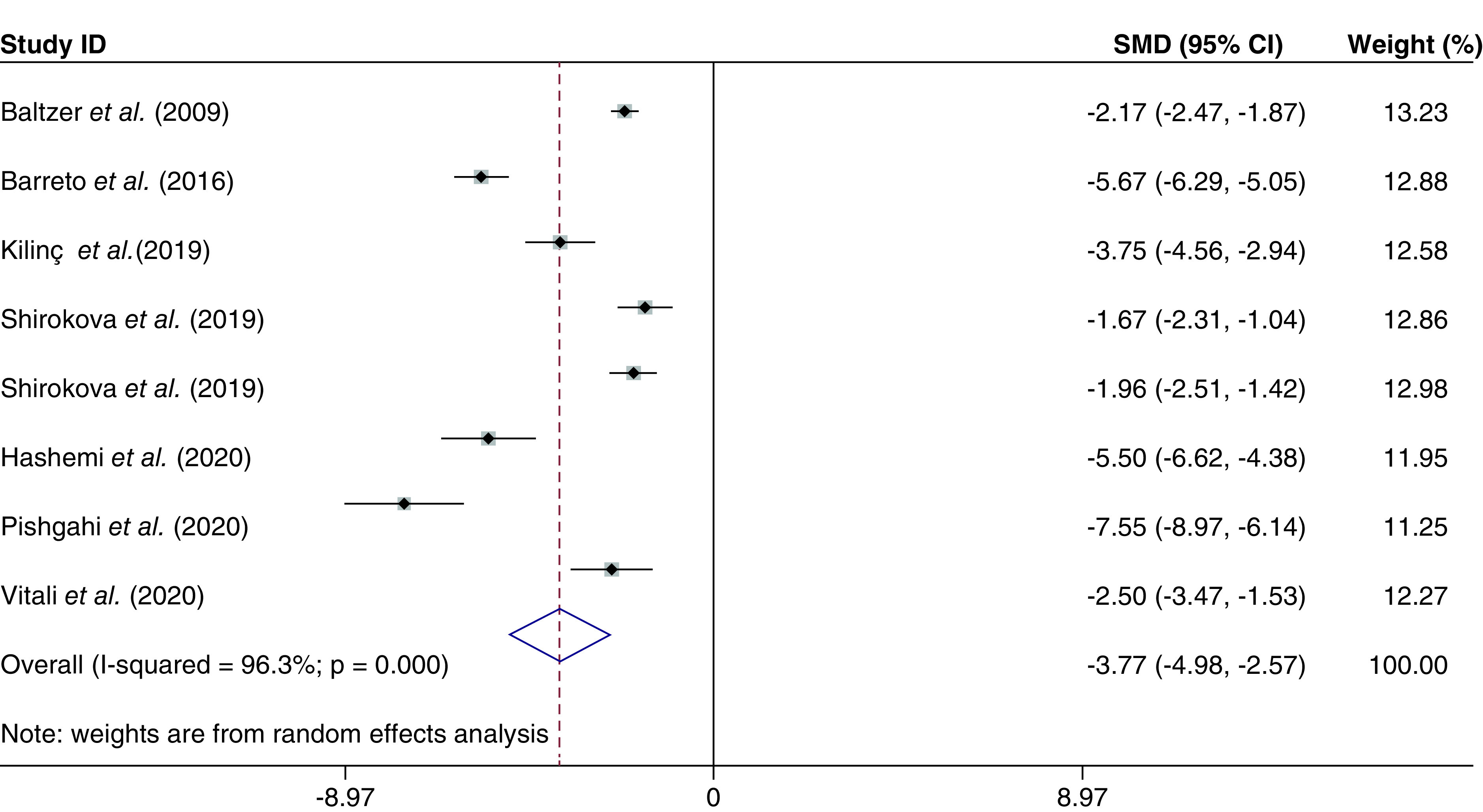
The visual analog scale score based on the random-effected model.

The VAS score was evaluated in seven studies [18,29,31–35], with a total of 409 individuals. The combined SMD for the VAS score was -3.77 (95% CI: -4.98 to -2.57; p < 0.000; I^2^ = 96.3%) (Figure 5). In addition, combined WMD was -32.37 (95% CI: -36.59 to -28.15; p < 0.000; I^2^ = 91.8%).

#### Risk of bias across studies

##### Publication bias

**Figure 6.**
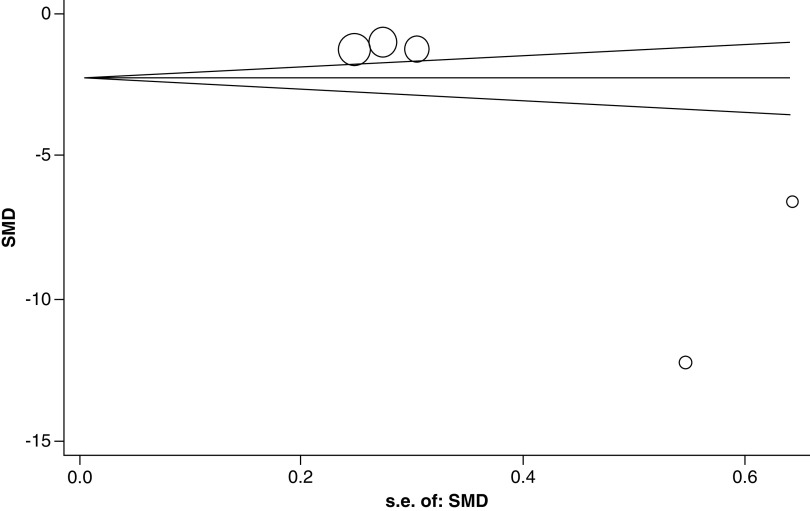
Begg’s funnel plot with pseudo 95% confidence limits. SMD: Standardized mean difference.

The publication bias was evaluated by the rank correlation analysis on SMDs (the Begg’s method). Based on its results, there was no evidence of publication bias in these studies (p = 0.142) (Figure 6); this means that studies with negative and positive results have been published.

#### Additional analysis

**Figure 7.**
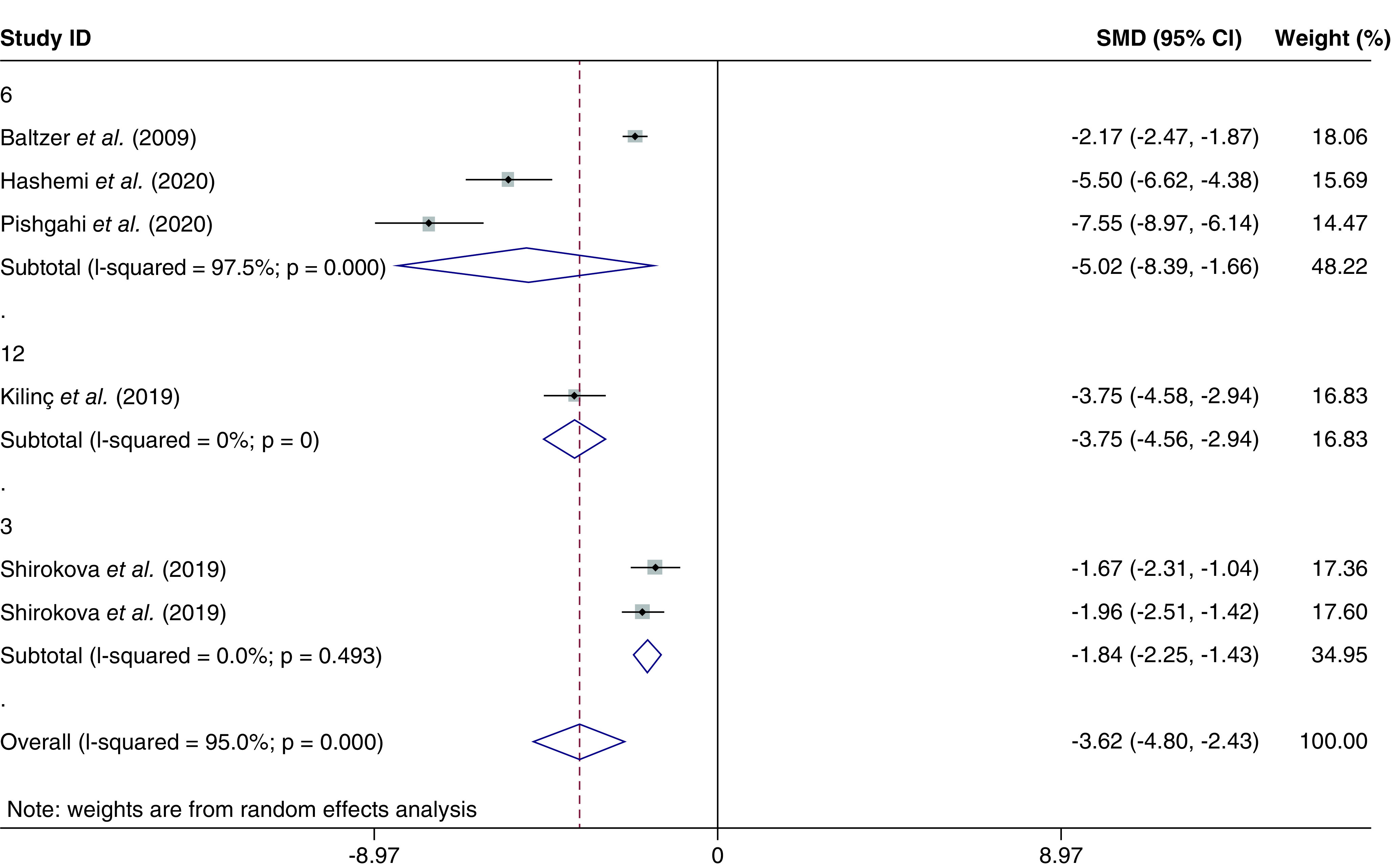
Forrest plot, subgroup analysis of Western Ontario and McMaster Universities score based on follow-up periods (standardized mean difference, 95% CI).

**Figure 8.**
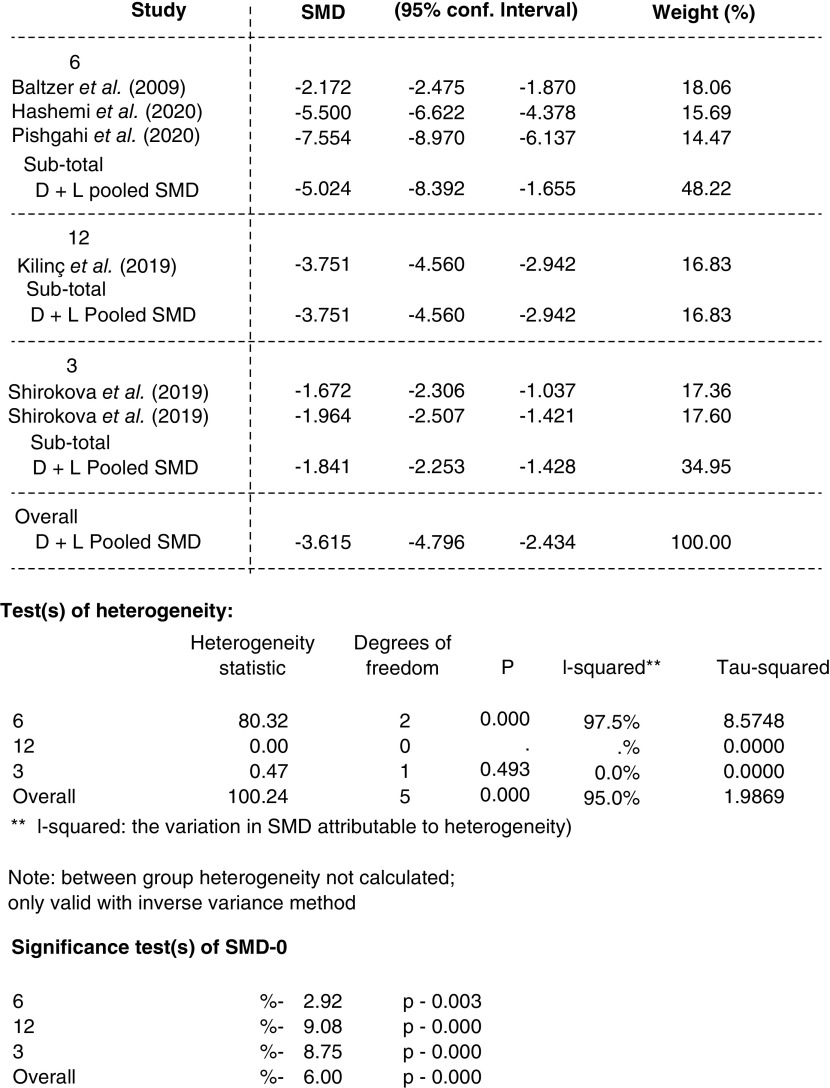
Subgroup analysis data of Western Ontario and McMaster Universities score based on follow-up periods.

Subgroup analysis for WOMAC score, SMD was performed based on the follow-up periods of different studies as applicable; Baltzer *et al.*, Pishgahi *et al.* and Hashemi *et al.* with 6 months follow up showed a more significant reduction in WOMAC during 6 months in Hashemi *et al.* and Pishgahi studies (p = 0.000). The two subgroups of the Shirokova *et al.*’s study with 3 months follow up showed less reduction in the WOMAC SMD relative to other studies (Figures 7 & 8).

## Discussion

The prevalence of OA, especially in the elderly, has led to many attempts to find curative treatments for this disease. In the meantime, several therapeutic guidelines have been provided by prominent academic associations [2]. However, due to the less-understood pathophysiology of this disease, current treatment options lead to symptomatic treatment and no curative treatment option has been provided [36].

Recent studies have introduced new regenerative treatments such as intra-articular injections of biological compounds such as hyaluronic acid, PRP, ozone, prolotherapy with dextrose, etc. that show their effectiveness to reduce pain and improve the patients’ physical function, which has been evaluated in various studies [37–40].

In the mid-1990s, after identifying the role of the inflammatory process in the pathophysiology of OA, ACS rich in endogenous anti-inflammatory cytokines such as the IL-1RAP was introduced and soon gained attention [19,41].

In 2008, Yang *et al.* examined the results of intra-articular injections of an ACS and normal saline (the control group) in 167 patients with knee OA and followed them for 12 months and found no difference in the improvement of WOMAC criteria between the intervention and placebo groups [26]. However, in the ACS group, the score of the KOOS was better than the other group, although this difference was not statistically significant. Therefore, this study did not recommend ACS as an effective treatment for OA [26].

One year later, Baltzer *et al.* provided evidence in favor of ACS efficacy in a double-blind clinical trial. The study was performed on 376 people (over 30 years old) with idiopathic knee OA (Kellgren–Lawrence Grade II–III) who had been symptomatic for more than 3 months.

Patients in the three groups underwent six injections of ACS or hyaluronic acid or normal saline (as a placebo) at a 6-month follow up. A significant improvement in the outcome measures was reported in the ACS group compared with the other intervention groups, without any side effects [29,42].

It is noteworthy that these researchers followed 310 of these patients over the next 2 years and re-reported the long-term efficacy and safety of ACS and its superiority to the control group [42].

Regarding the lasting effects of the ACS, the subgroup analysis of WOMAC scores based on follow-up periods showed the maximum efficacy in the 6 months follow up and a smaller reduction in the 3 and 12 months’ visits.

Fox *et al.* and Frizziero *et al.* in a separate review study on animal and human models identified ACS treatment as an effective and recommended method for knee OA [43,44].

On the other hand, a review study by Fortier *et al.*, examining the evidence for the effectiveness of growth factors on cartilage repair, showed that despite obtaining favorable clinical results, *in vitro* ACS administration had no significant effect on cartilage tissue repair, and further studies are needed to confirm this effect [45].

In 2015, an observational study was conducted by Baselga *et al.* In this study, 118 patients with knee OA underwent conservative treatment with ACS along with physiotherapy [25]. The results of this study showed that ACS injection caused a significant improvement in the WOMAC criteria and only one of these patients eventually required surgery.

As a relatively newly introduced method, comparing to PRP, ACS is different in some aspects, which comprises increased amounts of thrombocytes and growth factors such as VEGF, FGF, IGF-1 and IGF-2 and HGF. On the other hand, ACS is a cell-free product containing the IL-1Ra and a high concentration of IGF-1, HGF and FGF [46]. Moreover, regarding the cell-free nature of ACS, it is expected to cause less frequent allergic reactions in comparison to PRP and other similar blood products.

Based on the results of this meta-analysis, the intra-articular injection of ACS in patients with knee OA could decrease the global WOMAC score, and VAS score to a notable size, indicating that intra-articular injection of ACS can improve function in patients’ daily activities and reduce pain. This lends support to previous findings in the most literature. So ACS can be considered as an appropriate biologic agent for the treatment of patients with OA.

## Limitations

Despite acceptable results regarding publication bias, some factors are limiting this study. In the outcome level and study level, different outcome measures reported in some articles varied follow-up intervals and doses of injections, and also a number of studies with a high risk of bias were sources of limitation for this study. Heterogeneity between studies and the relatively small number of articles and sample sizes that were finally included, affected the quality of this review.

Above all, it is necessary to mention that performing a network meta-analysis would have been a better statistical approach to obtain more reliable results, although it requires recruiting eligible studies with a more homogenous methodology.

## Conclusion

Knee OA treatment is still a matter of controversy between expert authors as ACS can be an effective therapeutic choice, this study aimed to evaluate its efficacy with a systematic review and meta-analysis approach. The present study resulted in favor of the studied intervention, reporting that the intra-articular injection of ACS can reduce pain and improve function in patients’ daily activities (indicated by VAS and WOMAC outcome measures, respectively). Hence, ACS can be considered as an appropriate option for therapeutic goals of pain reduction and function improvement in patients with OA.

**Summary points**

- Knee osteoarthritis (OA) is a common and disabling disorder with no curative treatment yet.
- Regenerative medicine including platelet-rich plasma, plasma rich in growth factor and mesenchymal cells has gained attention recently, and autologous conditioned serum (ACS), which is a cell-free plasma containing anti-inflammatory components (mainly IL-1 receptor antagonist), has been studied and shown mostly promising but mixed results.
- The differences between ACS and platelet-rich plasma (as an accepted effective treatment) make it worthy of further evaluation. The less variable processing method and the theoretical potential of disease modification ability (as shown in some studies with animal subjects) and also the cell-free nature of this product (which reduced the risk of allergic reactions) are among the favorable characteristics of ACS.
- This study is aimed to gather the available information on this subject using a meta-analysis approach to obtain a conclusion on the efficacy of ACS in knee OA treatment.
- The meta-analysis resulted in a significant difference in the evaluated outcome measures (visual analog scale for pain and Western Ontario and McMaster Universities for function), the subgroup analysis also shown the most changes in the 6-month follow-up period (in comparison to 3 and 12 months).
- It suggested that ACS can reduce pain and improve function in these patients, with minimal adverse events reported.

## References

[1] Wehling P, Evans C, Wehling J, Maixner W. Effectiveness of intra-articular therapies in osteoarthritis: a literature review. Ther. Adv. Musculoskelet. Dis. 9(8), 183–196 (2017). 28835778 10.1177/1759720X17712695 PMC5557186

[2] Mora JC, Przkora R, Cruz-Almeida Y. Knee osteoarthritis: pathophysiology and current treatment modalities. J. Pain Res. 11, 2189–2196 (2018). 30323653 10.2147/JPR.S154002 PMC6179584

[3] Torrero JI, Martinez C. New developments in the treatment of osteoarthritis - focus on biologic agents. Open Access Rheumatol. 7, 33–43 (2015). 27790043 10.2147/OARRR.S50058 PMC5045124

[4] Raeissadat SA, Tabibian E, Rayegani SM, Rahimi-Dehgolan S, Babaei-Ghazani A. An investigation into the efficacy of intra-articular ozone (O2–O3) injection in patients with knee osteoarthritis: a systematic review and meta-analysis. J. Pain Res. 11, 2537 (2018). 30498370 10.2147/JPR.S175441 PMC6207244

[5] Tehrani-Banihashemi A, Davatchi F, Jamshidi AR, Faezi T, Paragomi P, Barghamdi M. Prevalence of osteoarthritis in rural areas of Iran: a WHO-ILAR COPCORD study. Int. J. Rheum. Dis. 17(4), 384–388 (2014). 24618176 10.1111/1756-185X.12312

[6] Litwic A, Edwards MH, Dennison EM, Cooper C. Epidemiology and burden of osteoarthritis. Br. Med. Bull. 105, 185–199 (2013). 23337796 10.1093/bmb/lds038 PMC3690438

[7] Hochberg MC, Altman RD, April KT American College of Rheumatology 2012 recommendations for the use of nonpharmacologic and pharmacologic therapies in osteoarthritis of the hand, hip, and knee. Arthritis Care Res. 64(4), 465–474 (2012). 10.1002/acr.21596 22563589

[8] Dohan Ehrenfest DM, Andia I, Zumstein MA, Zhang C-Q, Pinto NR, Bielecki T. Classification of platelet concentrates (Platelet-Rich Plasma-PRP, Platelet-Rich Fibrin-PRF) for topical and infiltrative use in orthopedic and sports medicine: current consensus, clinical implications, and perspectives. Muscles Ligaments Tendons J. 4(1), 3–9 (2014). 24932440 PMC4049647

[9] Jain K, Murphy PN, Clough TM. Platelet rich plasma versus corticosteroid injection for plantar fasciitis: a comparative study. Foot (Edinb.) 25(4), 235–237 (2015). 26362235 10.1016/j.foot.2015.08.006

[10] Raeissadat SA, Babaee M, Rayegani SM An overview of platelet products (PRP, PRGF, PRF, etc.) in the Iranian studies. Future Sci. OA 3(4), FSO231 (2017). 29134118 10.4155/fsoa-2017-0045 PMC5674219

[11] Raeissadat SA, Ghorbani E, Sanei Taheri M MRI changes after platelet rich plasma injection in knee osteoarthritis (randomized clinical trial). J. Pain Res. 13, 65–73 (2020). 32021396 10.2147/JPR.S204788 PMC6959502

[12] Raeissadat SA, Rayegani SM, Ahangar AG, Abadi PH, Mojgani P, Ahangar OG. Efficacy of intra-articular injection of a newly developed plasma rich in growth factor (PRGF) versus hyaluronic acid on pain and function of patients with knee osteoarthritis: a single-blinded randomized clinical trial. Clin. Med. Insights Arthritis Musculoskelet. Disord. 10, 1179544117733452 (2017). 29051707 10.1177/1179544117733452 PMC5638152

[13] Fotouhi A, Maleki A, Dolati S, Aghebati-Maleki A, Aghebati-Maleki L. Platelet rich plasma, stromal vascular fraction and autologous conditioned serum in treatment of knee osteoarthritis. Biomed. Pharmacother. 104, 652–660 (2018). 29803179 10.1016/j.biopha.2018.05.019

[14] O’Shaughnessy K, Matuska A, Hoeppner J Autologous protein solution prepared from the blood of osteoarthritic patients contains an enhanced profile of anti-inflammatory cytokines and anabolic growth factors. J. Orthop. Res. 32(10), 1349–1355 (2014). 24981198 10.1002/jor.22671 PMC4134723

[15] Frisbie DD. Autologous-conditioned serum: evidence for use in the knee. J. Knee Surg. 28(1), 63–66 (2015). 25599270 10.1055/s-0034-1543956

[16] Auw Yang KG, Raijmakers NJ, Van Arkel ER Autologous interleukin-1 receptor antagonist improves function and symptoms in osteoarthritis when compared to placebo in a prospective randomized controlled trial. Osteoarthr. Cartil. 16(4), 498–505 (2008). 10.1016/j.joca.2007.07.008 17825587

[17] Evans CH, Chevalier X, Wehling P. Autologous conditioned serum. Phys. Med. Rehabil. Clin. N. Am. 27(4), 893–908 (2016). 27788906 10.1016/j.pmr.2016.06.003

[18] Kılınç BE, Öç Y. Evaluation of the autologous conditioned serum in the treatment of osteoarthritis. Arch. Clin. Exp. Med. 4(2), 94–98 (2019).

[19] Arbel R. Orthokine. In: Bio-orthopaedics. Springer-Verlag Berlin Heidelberg, Germany, 561–569 (2017).

[20] Tassara M, De Ponti A, Barzizza L Autologous conditioned serum (ACS) for intra-articular treatment in osteoarthritis: retrospective report of 28 cases. Transfus. Apher. Sci. 57(4), 573–577 (2018). 30131208 10.1016/j.transci.2018.07.021

[21] Rutgers M, Creemers LB, Auw Yang KG, Raijmakers NJ, Dhert WJ, Saris DB. Osteoarthritis treatment using autologous conditioned serum after placebo. Acta Orthop. 86(1), 114–118 (2015). 25140983 10.3109/17453674.2014.950467 PMC4366668

[22] Shirokova K, Noskov S, Shirokova L. Comparison of clinical efficacy of platelet-rich plasma and autologous conditioned serum treatment in patients with osteoarthritis of the knee. Osteoarthr. Cartil. 25, S438 (2017).

[23] Liberati A, Altman DG, Tetzlaff J The PRISMA statement for reporting systematic reviews and meta-analyses of studies that evaluate health care interventions: explanation and elaboration. J. Clin. Epidemiol. 62(10), e1–e34 (2009). 19631507 10.1016/j.jclinepi.2009.06.006

[24] Higgins JPT, Altman DG, Sterne JAC. Assessing risk of bias in included studies. In: Cochrane Handbook for Systematic Reviews of Interventions. Higgins JPT, Green S John Wiley & Sons Ltd.West Sussex, UK, 194–206 (2011).

[25] Baselga Garcia-Escudero J, Miguel Hernandez Trillos P. Treatment of osteoarthritis of the knee with a combination of autologous conditioned serum and physiotherapy: a two-year observational study. PLoS ONE 10(12), e0145551 (2015). 26709697 10.1371/journal.pone.0145551 PMC4692499

[26] Yang KA, Raijmakers N, Van Arkel E Autologous interleukin-1 receptor antagonist improves function and symptoms in osteoarthritis when compared to placebo in a prospective randomized controlled trial. Osteoarthr. Cartil. 16(4), 498–505 (2008). 10.1016/j.joca.2007.07.008 17825587

[27] Zarringam D, Bekkers JE, Saris DB. Long-term effect of injection treatment for osteoarthritis in the knee by Orthokin autologous conditioned serum. Cartilage 9(2), 140–145 (2018). 29172669 10.1177/1947603517743001 PMC5871127

[28] Rutgers M, Creemers LB, Yang KGA, Raijmakers NJ, Dhert WJ, Saris DB. Osteoarthritis treatment using autologous conditioned serum after placebo: patient considerations and clinical response in a non-randomized case series. Acta Orthopaedica 86(1), 114–118 (2015). 25140983 10.3109/17453674.2014.950467 PMC4366668

[29] Baltzer A, Moser C, Jansen S, Krauspe R. Autologous conditioned serum (Orthokine) is an effective treatment for knee osteoarthritis. Osteoarthr. Cartil. 17(2), 152–160 (2009). 10.1016/j.joca.2008.06.014 18674932

[30] Fathalla M, Abd-El Motaal F, Abdulkareem O, Elganzoury A. Low-dose intra-articular autologous conditioned serum in treatment of primary knee osteoarthritis. Egypt. Rheumatol. Rehabil. 41(3), 98 (2014).

[31] Shirokova L, Noskov S, Gorokhova V, Reinecke J, Shirokova K. Intra-articular injections of a whole blood clot secretome, autologous conditioned serum, have superior clinical and biochemical efficacy over platelet-rich plasma and induce rejuvenation-associated changes of joint metabolism: a prospective, controlled open-label clinical study in chronic knee osteoarthritis. Rejuv. Res. 23(5), 401–410 (2019). 10.1089/rej.2019.2263 31847701

[32] Hashemi M, Adlkhoo H, Dadkhah P, Rohanifar R, Taheri M. A comparative assessment of autologous conditioned serum and ozone for knee osteoarthritis treatment: mid-term follow up. Novelty Biomed. 8(1), 45–52 (2020).

[33] Pishgahi A, Abolhasan R, Shakouri SK Effect of dextrose prolotherapy, platelet rich plasma and autologous conditioned serum on knee osteoarthritis: a randomized clinical trial. Iran J. Allergy Asthma Immunol. 19(3), 243–252 (2020). 32615658 10.18502/ijaai.v19i3.3452

[34] Vitali M, Ometti M, Drossinos A, Pironti P, Santoleri L, Salini V. Autologous conditioned serum: clinical and functional results using a novel disease modifying agent for the management of knee osteoarthritis. J. Drug Assess. 9(1), 43–51 (2020). 32284907 10.1080/21556660.2020.1734009 PMC7144201

[35] Barreto A, Braun TR. A new treatment for knee osteoarthritis: clinical evidence for the efficacy of Arthrokinex™ autologous conditioned serum. J. Orthop. 14(1), 4–9 (2017). 27821994 10.1016/j.jor.2016.10.008 PMC5090235

[36] Imani F, Patel VB. Therapeutic challenges for knee osteoarthritis. Anesth. Pain. Med. 9(3),e95377 (2019). 31497526 10.5812/aapm.95377 PMC6712428

[37] Jones IA, Togashi R, Wilson ML, Heckmann N, Vangsness CT. Intra-articular treatment options for knee osteoarthritis. Nat. Rev. Rheumatol. 15(2), 77–90 (2019). 30498258 10.1038/s41584-018-0123-4 PMC6390843

[38] Marc J-F. Regenerative medicine in osteoarthritis-a new chance for knee osteoarthritis patients. Int. J. Clin. Rheumatol. 13(5), 278–279 (2018).

[39] Duymus TM, Mutlu S, Dernek B, Komur B, Aydogmus S, Kesiktas FN. Choice of intra-articular injection in treatment of knee osteoarthritis: platelet-rich plasma, hyaluronic acid or ozone options. Knee Surg. Sports Traumatol. Arthrosc. 25(2), 485–492 (2017). 27056686 10.1007/s00167-016-4110-5

[40] Hashemi M, Jalili P, Mennati S The effects of prolotherapy with hypertonic dextrose versus prolozone (intraarticular ozone) in patients with knee osteoarthritis. Anesth. Pain. Med. 5(5), e27585 (2015). 26587401 10.5812/aapm.27585 PMC4644302

[41] Evans CH, Chevalier X, Wehling P. Autologous conditioned serum. Phys. Med. Rehabil. Clin. 27(4), 893–908 (2016). 10.1016/j.pmr.2016.06.003 27788906

[42] Moser C, Baltzer A, Jansen S, Krauspe R, Wehling P. 431. Efficacy of autologous conditioned serum (ACS-Orthokine) in osteoarthritis of the knee at two-year follow-up. The German Orthikine Osteoarthritis Trial-GOAT. Osteoarthr. Cartil. 15, C233 (2007).

[43] Fox BA, Stephens MM. Treatment of knee osteoarthritis with Orthokine-derived autologous conditioned serum. Expert Rev. Clin. Immunol. 6(3), 335–345 (2010). 20441419 10.1586/eci.10.17

[44] Frizziero A, Giannotti E, Oliva F, Masiero S, Maffulli N. Autologous conditioned serum for the treatment of osteoarthritis and other possible applications in musculoskeletal disorders. Br. Med. Bull. 105, 169–184 (2013). 22763153 10.1093/bmb/lds016

[45] Fortier LA, Barker JU, Strauss EJ, Mccarrel TM, Cole BJ. The role of growth factors in cartilage repair. Clin. Orthop. Relat. Res. 469(10), 2706–2715 (2011). 21403984 10.1007/s11999-011-1857-3 PMC3171543

[46] Genç E, Yüksel S, Çağlar A, Beytemur O, Güleç MA. Comparison on effects of platelet-rich plasma versus autologous conditioned serum on Achilles tendon healing in a rat model. Acta Orthop. Traumatol. Turc. 54(4), 438–444 (2020). 32812877 10.5152/j.aott.2020.18498 PMC7444885

